# Implication of Pappalysins and Stanniocalcins in the Bioavailability of IGF-I in Children With Type 1 Diabetes Mellitus

**DOI:** 10.1210/jendso/bvae081

**Published:** 2024-04-23

**Authors:** María Güemes, Álvaro Martín-Rivada, Beatriz Corredor, Patricia Enes, Sandra Canelles, Vicente Barrios, Jesús Argente

**Affiliations:** Department of Pediatrics & Pediatric Endocrinology, Hospital Infantil Universitario Niño Jesús, Research Institute La Princesa, 28009 Madrid, Spain; Department of Pediatrics & Pediatric Endocrinology, Hospital Infantil Universitario Niño Jesús, Research Institute La Princesa, 28009 Madrid, Spain; Department of Pediatrics & Pediatric Endocrinology, Hospital Infantil Universitario Niño Jesús, Research Institute La Princesa, 28009 Madrid, Spain; Department of Pediatrics & Pediatric Endocrinology, Hospital Infantil Universitario Niño Jesús, Research Institute La Princesa, 28009 Madrid, Spain; Department of Pediatrics & Pediatric Endocrinology, Hospital Infantil Universitario Niño Jesús, Research Institute La Princesa, 28009 Madrid, Spain; Department of Pediatrics & Pediatric Endocrinology, Hospital Infantil Universitario Niño Jesús, Research Institute La Princesa, 28009 Madrid, Spain; Centro de Investigación Biomédica en Red de Fisiopatología de la Obesidad y Nutriciόn (CIBEROBN), Instituto de Salud Carlos III, 28029 Madrid, Spain; Department of Pediatrics & Pediatric Endocrinology, Hospital Infantil Universitario Niño Jesús, Research Institute La Princesa, 28009 Madrid, Spain; Centro de Investigación Biomédica en Red de Fisiopatología de la Obesidad y Nutriciόn (CIBEROBN), Instituto de Salud Carlos III, 28029 Madrid, Spain; Department of Pediatrics, Universidad Autónoma de Madrid, 28029 Madrid, Spain; IMDEA, Food Institute, CEIUAM+CSI, 28049 Madrid, Spain

**Keywords:** type 1 diabetes mellitus, growth, IGF-I, IGFBP, pappalysin, stanniocalcin, children

## Abstract

**Context:**

Anomalies in the growth hormone (GH)/insulin-like growth factor (IGF) axis, are common in children with type 1 diabetes mellitus (T1DM), even in those reaching a normal or near-normal final height. However, concentrations of the IGF bioavailability regulatory factors (pappalysins [PAPP-As] and stanniocalcins [STCs]) have not been reported in children with T1DM.

**Objective:**

To determine serum concentrations of PAPP-As and STCs in children at diagnosis of T1DM and after insulin treatment and the correlation of these factors with other members of the GH/IGF axis, beta-cell insulin reserve, auxology, and nutritional status.

**Methods:**

A single-center prospective observational study including 47 patients (59.5% male), with T1DM onset at median age of 9.2 years (interquartile range: 6.3, 11.9) was performed. Blood and anthropometric data were collected at diagnosis and after 6 and 12 months of treatment.

**Results:**

At 6 and 12 months after T1DM diagnosis, there was improvement in the metabolic control (decrease in glycated hemoglobin [HbA1c] at 12 months −3.66 [95% CI: −4.81, −2.05], *P* = .001), as well as in body mass index SD and height SD (not statistically significant). STC2 increased (*P* < .001) and PAPP-A2 decreased (*P* < .001) at 6 and 12 months of treatment onset (*P* < .001), which was concurrent with increased total IGF-I and IGF-binding protein concentrations, with no significant modification in free IGF-I concentrations. HbA1c correlated with PAPP-A2 (*r* = +0.41; *P* < .05) and STC2 (*r* = −0.32; *P* < .05).

**Conclusion:**

Implementation of insulin treatment after T1DM onset modifies various components of the circulating IGF system, including PAPP-A2 and STC2. How these modifications modulate linear growth remains unknown.

Although significant advancements in insulin therapy for children with type 1 diabetes mellitus (T1DM) have allowed many patients to reach normal or just slightly reduced final height, anomalies of growth often persist [1]. Suboptimal prepubertal and pubertal growth likely reflects the duration of the disease and metabolic control [2, 3], as well as the impact of inflammatory markers, including interleukin-6, C-reactive protein, and fibrinogen, that directly affect the growth plate and suppress local insulin-like growth factor I (IGF-I) actions [4, 5]. Uncontrolled celiac disease and potentially, the recently popular very low-carbohydrate diets if not carefully managed [6], can also affect growth. To ensure physiological growth, normal insulin secretion and, especially, its portal concentrations, is indispensable for normal circulating concentrations of IGF-I and IGF-binding proteins (IGFBPs) [7]. Insulin is also reported to influence hepatic expression of the growth hormone (GH) receptor and to participate in post-receptor GH signaling, which influences IGF-I and IGFBP synthesis [7]. Low IGF-I concentrations lead to a reduction in its negative feedback on the pituitary, which is involved in the observed GH hypersecretion [8]. Exogenous insulin administered subcutaneously, or even via continuous infusion, is not able to ameliorate portal hepatic hypoinsulinization [9, 10]. Insufficient intraportal insulin in children with T1DM results in low circulating concentrations of IGF-I and IGFBP-3, as well as high IGFBP-1 [11] and GH concentrations [8]. Additionally, high IGFBP-1 levels could also inhibit IGF-I bioactivity [11].

The growth regulatory factors (pappalysins [pregnancy-associated plasma proteins, PAPP-A and PAPP-A2] and stanniocalcins [STC1 and STC2]) modulate the bioavailability of IGFs by regulating the concentrations of intact and free IGFBPs. In the circulation, IGFBPs bind IGF-I or IGF-II and acid-labile subunit (ALS) forming trimolecular complexes, and thus antagonizing the binding of free forms to the receptor. Pappalysins cleave IGFBPs, and hence can augment IGF bioactivity and subsequently enhance local and systemic IGF signaling [12]. The main target of PAPP-A is IGFBP-4 [13], but it can also cleave IGFBP-5 [14] and IGFBP-2 [15] to a lesser extent. Alternatively, PAPP-A2 cleaves IGFBP-3, as well as IGFBP-5 [14], and IGFBP-2 [16], and is considered one of the principal regulators of IGF-I bioavailability [17]. Stanniocalcins are glycoproteins known to inhibit the action of pappalysins [18, 19].

Concentrations of pappalysins and stanniocalcins have not yet been determined in children with T1DM, albeit their study in adults with diabetes has recently gained interest [20-22]. Stanniocalcins are expressed in the pancreatic islets, where STC1 colocalizes with insulin in beta-cells [20] and STC2 with glucagon in alpha-cells [21], where they are suggested to exert effects on glucose homeostasis and to be markers of the appearance and progression of diabetes [21, 22]. In a 20-year longitudinal study with 1506 participants, after multivariable selection, PAPP-A was the only protein associated with the onset of prediabetes and type 2 diabetes (T2DM), with associations still observed at the 20-year visit [23]. Another study in adults with T2DM, following multivariable adjustment, found that higher concentrations of STC2, PAPP-A, and both intact and total IGFBP-4 associated with all-cause mortality [24]. Conversely, other studies have not found a consistent association between PAPP-A and T2DM [25-27].

With the aim to better characterize the physiology of growth in children with T1DM, this study investigated: (i) the baseline serum concentrations of pappalysins and stanniocalcins upon diagnosis; (ii) the effect of insulin treatment on these factors; and (iii) the possible correlation with members of the GH/IGF axis, beta-cell insulin reserve, auxology, and nutrition status.

## Methods

### Ethics Statement

This study was approved by the ethical committee of Hospital Infantil Universitario Niño Jesús, and it adheres to the ethical principles of the Declaration of Helsinki.

### Patients and Study Design

**Figure 1.**
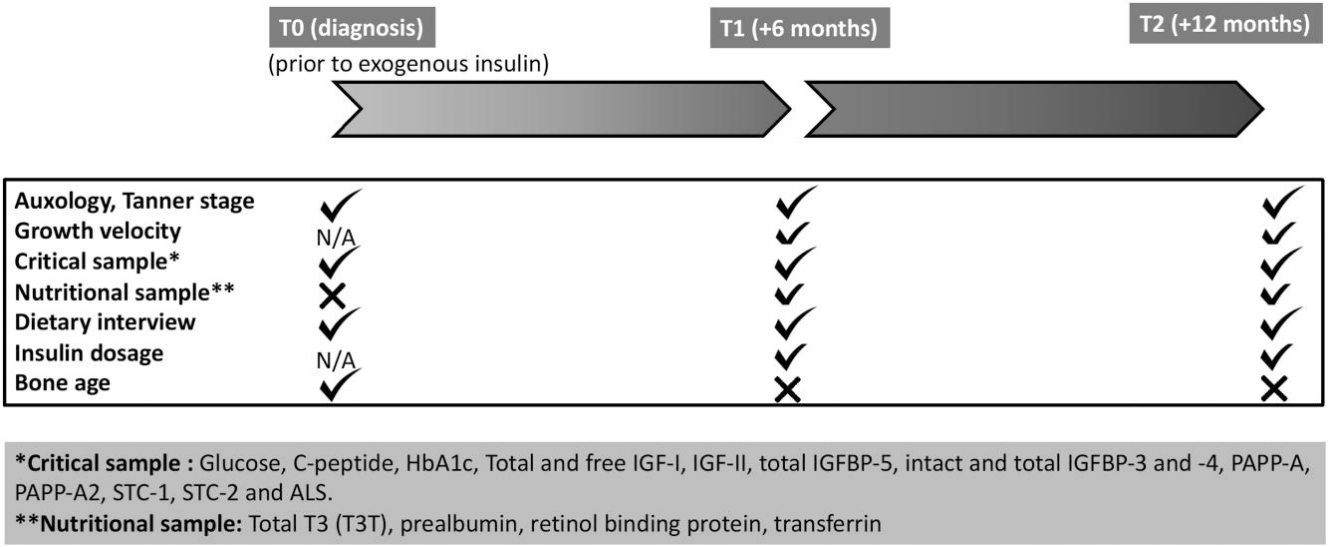
Flowchart of data collection. Checkmark represents timepoints when samples/measurements were taken. X represents timepoints without such information. Abbreviation: N/A, not available or not applicable.

This single-center prospective observational study was carried out in pediatric patients (age < 18 years) diagnosed with T1DM. Positive pancreatic autoimmunity was an inclusion criterion. Forty-seven children were assessed between November 2019 and November 2021. The study was carried out in 3 stages: upon diagnosis during hospitalization (T0), at 6 months after diagnosis (T1), and at 12 months after diagnosis (T2). See Fig. 1 where the flowchart of data collection is depicted.

Demographics (sex, date of birth) and medical conditions were recorded. Patients were clinically evaluated at the endocrinology department where weight, body mass index (BMI) and standing height measurements were performed at T0, T1, and T2, with growth velocity being assessed at T1 and T2, and standardized according to Spanish normative data [28]. Pubertal status was assessed by Tanner staging.

### Serum Analysis

Fasting blood samples were extracted at T0, T1, and T2. Total and free IGF-I, IGF-II, total IGFBP-2 and IGFBP-5, intact and total IGFBP-3 and IGFBP-4, PAPP-A, and STC2 were determined by using commercial enzyme-linked immunosorbent assay (ELISA) kits (Ansh Labs, Webster, TX, USA). STC1 was measured by ELISA (R&D Systems, Minneapolis, MN, USA). ALS was measured by ELISA (Mediagnost, Reutlingen, Germany), and PAPP-A2 was determined by a chemiluminescence immunoassay (Cloud Clone, Katy, TX, USA). Research Resource Identifiers for the immunoassays employed in this study are available in Table 1. All data of the IGF axis were standardized for sex and pubertal development, according to recent normative data [29]. Capillary glycated hemoglobin (HbA1c) was analyzed in the outpatient clinic (DCA Vantage Analyzer Class 1, Siemens Healthcare Diagnostics Ltd, Camberley, UK). Glucose, insulin (this parameter was only analyzed at T0), C-peptide, total triiodothyronine (T3T), transferrin, prealbumin, and retinol binding protein were analyzed in the hospital's validated laboratory. Metabolic control determined by means of time in range as measured by a glucose sensor was unavailable at the time in many patients, and thus, not included in this study.

**Table 1.** Research Resource Identifier (RRID) of the assays used

Parameters	Commercial source	Catalog #	RRID
Total IGF-I	Ansh Labs	AL-121	AB_2783672
Free IGF-I	Ansh Labs	AL-122	AB_2783673
IGF-II	Ansh Labs	AL-131	AB_2783680
IGFBP-2	Ansh Labs	AL-140	AB_2783686
Total IGFBP-3	Ansh Labs	AL-120	AB_2783671
Intact IGFBP-3	Ansh Labs	AL-149	AB_2783688
Total IGFBP-4	Ansh Labs	AL-126	AB_2783676
Intact IGFBP-4	Ansh Labs	AL-128	AB_2783678
IGFBP-5	Ansh Labs	AL-127	AB_2783677
ALS	Mediagnost	E35	AB_2813809
Insulin	BioVendor	RIS006R	AB_2893123
PAPP-A	Ansh Labs	AL-101	AB_2783656
PAPP-A2	Cloud Clone	SCD471Hu	AB_2893124
STC-1	R&D Systems	DY2958	AB_2893122
STC-2	Ansh Labs	AL-143	AB_2783687

Abbreviations: ALS, acid-labile subunit; IGF, insulin-like growth factor; IGFBP-2, insulin-like growth factor binding protein-2; IGFBP-3, insulin-like growth factor binding protein-3; IGFBP-4, insulin-like growth factor binding protein-4; IGFBP-5, insulin-like growth factor binding protein-5; PAPP-A, pregnancy-associated plasma protein A; PAPP-A2, pregnancy-associated plasma protein A2; STC-1, stanniocalcin 1; STC-2, stanniocalcin 2.

### Statistics

Quantitative variables are expressed as median and interquartile range (IQR). Qualitative variables are expressed as absolute and relative frequencies. To check for normality, the Shapiro-Wilk test was used. Differences between groups were investigated with ANOVA for repeated measures. Logarithmic transformation was used to transform skewed variables into a normalized dataset. The Fisher correlation test was performed to determine whether there was linear association between quantitative variables. The statistical analysis was performed with STATA 15.1. Results were considered statistically significant when the *P* value was below .05.

## Results

### Patients

The median age of diabetes onset was 9.16 years (IQR: 6.30, 11.91), with 28 male (59.5%) and 19 female youths (40.4%). They were all previously healthy children except for 3 cases with autoimmune thyroiditis, 1 with celiac disease (adhering to gluten-free diet at diagnosis of T1DM and throughout this study) and 1 case with 17q12 microdeletion. None of them were receiving chronic medications while in this study. Twelve participants (25.5%) had a positive family history of T1DM. Autoantibodies (anti-GAD +/− anti-insulin +/− anti-IA2 +/− ICA) were positive in all cases.

### Metabolic Control, Growth, and Nutrition

Regarding the onset of the disease, 16 children (34%) presented with severe ketoacidosis, 9 (19.1%) with moderate ketoacidosis, 9 (19.1%) with mild ketoacidosis, and 13 (27.6%) with hyperosmolar hyperglycemia. The median HbA1c level at diagnosis was 11.2% (IQR: 9.8, 13.1). At T1, median C-peptide was 0.23 ng/mL (IQR: 0.17, 0.57) and HbA1c 7.2% (IQR: 6.7, 8.2). At T2, median C-peptide was 0.16 ng/mL (IQR: 0.05, 0.41) and HbA1c 7.6% (IQR: 6.8, 8.5). There was a statistically significant improvement in HbA1c from T0 to T1 and T2 (Table 2), whereas no statistical significance was found in C-peptide change.

**Table 2.** Comparison of HbA1c, height SD, and BMI SD at T0, T1, and T2

Variable	T1 vs T0	T2 vs T0	*P*	*R^2^* adjusted
HbA1c	−3.99 (95% CI: −4.94, −3.03) *P* < .001	−3.66 (95% CI: −4.81, −2.05) *P* < .001	.0001***	0.5187
Height SD	−0.23 (95% CI: −0.74, 0.26) *P* = .350	−0.18 (95% CI: −0.79, 0.43) *P* = .565	.6113	−0.0129
BMI SD	0.13 (95% CI: −0.46, 0.71) *P* = .668	0.30 (95% CI: −0.42, 1.02) *P* = .411	.6962	−0.0164

Abbreviations: BMI, body mass index; HbA1c, glycated hemoglobin; m: months. ***P < 0.001

At the time of diagnosis of T1DM (T0), the median patient height was −0.44 SD (IQR: −0.35, 1.05), weight was −0.1 SD (IQR: −0.64, 0.54), BMI was −0.27 SD (IQR: −1.11, 0.20) and Tanner stages were: I (29/47); II (6/47); III (5/47); IV (2/47), and V (5/47). At T1, median height was 0.05 SD (IQR: −0.30, 0.86), weight −0.12 SD (IQR: −0.62, 0.42), and BMI −0.03 SD (IQR: −0.74, 0.3). At T2, median height was 0.0 SD (IQR: −0.10, 1.04), weight −0.10 SD (IQR: −0.67, 0.76), and BMI 0.0 SD (IQR: −0.78, 0.3). There were no statistical differences between height, weight, and BMI SD from T0 to T1 and T2 (Table 2). Growth velocity SD at T1 was 0.80 (IQR: −0.40, 1.79) and at T2 0.70 (IQR: 0.33, 1.00), with no statistically significant difference (Table 3).

**Table 3.** Comparison of growth velocity and nutritional markers between T1 and T2

Variable	T2 vs T1	*P*	*R^2^* adjusted
GV SD	0.23 (95% CI: −0.72, 1.19)	.6245	−0.0234
Prealbumin	1.82 (95% CI: −0.37, 4.00)	.1001	0.0660
Transferrin	30.91 (95% CI: 0.36, 61.47)	.0476*	0.1348
Retinol	−0.00 (95% CI: −0.20, 0.20)	.9769	−0.0384

Abbreviation: GV, growth velocity. *P < .05

All patients confirmed that they followed a varied Mediterranean diet prior and during this study. Although nutritional markers were not extracted at T0, available data at T1 shows a median transferrin of 244 mg/dL (IQR: 219, 263), prealbumin 16.8 mg/dL (IQR: 16.1, 19), retinol binding globulin 1.32 mg/dL (IQR: 1.06, 1.51) and T3T 1.36 ng/mL (IQR: 1.26, 1.49). At T2, median transferrin was 267 mg/dL (IQR: 258, 284), prealbumin 18.6 mg/dL (IQR: 17.0, 20.8), retinol binding globulin 1.26 mg/dL (IQR: 1.15, 1.41) and T3T 1.38 ng/mL (IQR: 1.23, 1.51). Transferrin was the only nutritional marker which significantly increased from T1 to T2 (Table 3).

### Serum Concentrations of the GH/IGF Axis

**Figure 2.**
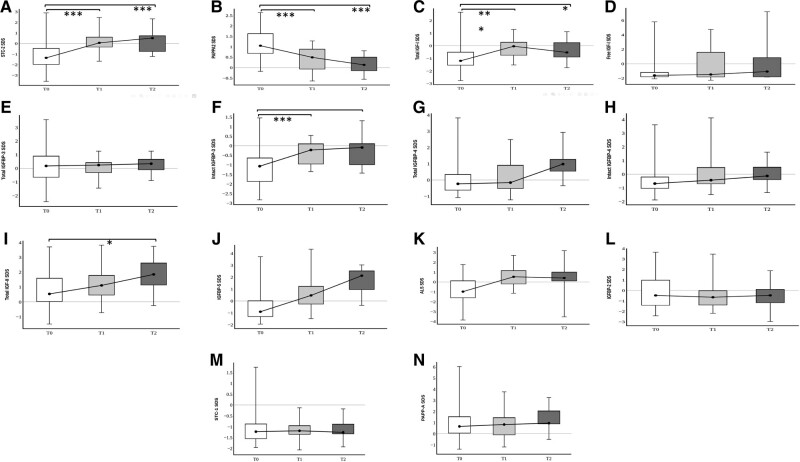
Boxplot representation of serum concentrations of total and free or intact forms of IGFs and IGFBPs, pappalysins, stanniocalcins, and ALS, at times T0, T1, and T2. A, STC2 SDS; B, PAPP-A2 SDS; C, Total IGF-I SDS; D, Free IGF-I SDS; E, Total IGFBP-3 SDS; F, Intact IGFBP-3 SDS; G, Total IGFBP-4 SDS; H, Intact IGFBP-4 SDS. I, IGF-II SDS; J, IGFBP-5 SDS; K, ALS SDS; L, IGFBP-2 SDS; M, STC1 SDS; N, PAPP-A SDS. *P < .05, ** *P* < .01, ***P < .001.

All serum concentrations of pappalysins, stanniocalcins, free and total IGF-I, IGF-II, and total and intact IGFBPs are presented in Fig. 2 and Table 4. There were no significant differences in concentrations between both sexes. All analyte concentrations remained within the limits of normality (−2.0, +2.0 SD) during this study, except for an outlier value for total IGF-II at T2 [median: 1.71 SD (IQR: 1.08, 2.41]) and IGFBP-5 at T2 (1.95 SD [0.72, 2.42]). However, upon diagnosis, STC2 concentrations were in the lower part of normality (−1.31 SD [−1.97, −0.39]) and significantly increased after 6 and 12 months of insulinization (0.07 SD [−0.37, 0.69], *P* < .001 and 0.52 SD [(−0.76, 0.90], *P* < .001, respectively). The STC2 increase was paralleled by a significant decrease in PAPP-A2. PAPP-A2 concentrations were in the upper part of normality at diagnosis (1.05 SD [0.68, 1.63]), and significantly decreased during insulinization (6 months: 0.49 SD [−0.07, 0.92], *P* < .001; 12 months: 0.13 SD [−0.16, 0.52], *P* < .001). STC1 and PAPP-A concentrations were within their normal range at diagnosis and remained unvaried throughout the study. Total and free IGF-I SD and IGFBP-3 concentrations increased during insulin treatment, but they remained within the normal range at all times. There was also an increase in total IGF-II, total IGFBP-3, total IGFBP-4, IGFBP-5, as well as in intact IGFBP-3, intact IGFBP-4, ALS, and PAPP-A over time. IGFBP-2 concentrations remained unvaried.

**Table 4.** Serum concentrations of pappalysins, stanniocalcins, ALS, total and intact IGF, and IGFBPs

Analyte (SD)	T0 [median (IQR)]	T1 [median (IQR)]	T2 [median (IQR)]
Total IGF-I	−1.20 (−1.56, −0.50)	−0.04 (−0.77, 0.3)***	−0.53 (−0.89, 0.25)^#^
Free IGF-I	−1.59 (−1.78, −1.18)	−1.56 (−1.8, −0.97)	−1.29 (−1.8, 0.65)
Total IGF-II	0.54 (0.01, 1.68)	1.08 (0.39, 1.63)	1.71 (1.08, 2.41)^#^
ALS	−0.94 (−1.59, 0.15)	0.53 (−0.24, 1.18)***	0.41 (0.11, 1.01)^##^
IGFBP-2	−0.48 (−1.42, 0.49)	−0.83 (−1.43, −0.07)	−0.47 (−1.19, 0.12)
Total IGFBP-3	0.15 (−0.71, 0.88)	0.24 (−0.32, 0.44)	0.34 (−0.13, 0.73)
Intact IGFBP-3	−1.07 (−1.89, −0.62)	−0.22 (−0.97, 0.11)***	−0.09 (−1.01, 0.15)^##^
Total IGFBP-4	−0.24 (−0.64, 0.24)	−0.16 (−0.58, 0.97)	0.98 (0.45, 1.32)^###^
Intact IGFBP-4	−0.72 (−1.04, −0.23)	−0.47(−0.73, 0.45)	−0.13 (−0.47, 0.65)
IGFBP-5	−0.93 (−1.35, −0.03)	0.45 (−1.8, −0.97)***	1.95 (0.72, 2.42)^###^
STC1	−1.23 (−1.56, −0.84)	−1.19 (−1.37, −0.94)	−1.26 (−1.35, −0.85)
STC2	−1.31 (−1.97, −0.39)	0.07 (−0.37, 0.69)***	0.52 (−0.76, 0.90)^###^
PAPP-A	0.63 (−0.08, 1.33)	0.80 (−0.3, 1.27)	0.93 (0.76, 1.92)
PAPP-A2	1.05 (0.68, 1.63)	0.49 (−0.07, 0.92)***	0.13 (−0.16, 0.52)^###^

ANOVA *P* value of T0 vs T1 (*P < .05, ** *P* < .01, ***P < .001) and T0 vs T2 (#*P* < .05, ## *P* < .01, ###*P* < .001). Abbreviations: ALS, acid-labile subunit; IGF, insulin-like growth factor; IGFBP, insulin-like growth factor binding protein; IQR, interquartile range; PAPP-A, pregnancy-associated plasma protein A; PAPP-A2, pregnancy-associated plasma protein A2; STC, stanniocalcin.

When investigating for differences between variables depending on Tanner stage (prepubertal vs pubertal stages), total IGF-II showed statistical change in prepubertal children (Delta mean T1 vs T0: 0.97 [95% CI −0.35, 2.28], *P* = .026; Delta mean T2 vs T0 2.22 [95% CI 0.58, 3.85] *P* = .009). No other variable showed statistical difference between prepubertal and pubertal stage.

### Correlations

Correlations were examined between pappalysins, stanniocalcins, and markers of the growth axis (IGFs, IGFBPs), beta-cell reserve (C-peptide), initial presentation (ketoacidosis, hyperglycemia), HbA1c, exogenous insulin dose, auxology (height, growth velocity, weight, BMI), and nutrition (prealbumin, transferrin, T3T, and retinol). Significant correlations are collected in Table 5. There were no significant correlations found with growth velocity, C-peptide, initial presentation, and insulin dose.

**Table 5.** Correlations

HbA1c	PAPP-A2 (*r*: 0.41), STC2 (*r*: −0.32), intact IGFBP-3 (*r*: −0.29), transferrin (*r*: 0.45), prealbumin (*r*: −0.38)
PAPP-A	STC1 (*r*: −0.27), free IGF-I (*r*: 0.35), total IGF-II (*r*: 0.29)
STC1	ALS (*r*: −0.28), total IGF-2 (*r*: −0.23), intact IGFBP-4 (*r*: 0.26)
STC2	STC1 (*r*: −0.23), ALS (*r*: 0.37), total IGF-2 (*r*: 0.34), prealbumin (*r*: 0.64)
Free IGF-I	STC2 (*r*: 0.29), ALS (*r*: 0.25), total IGF-II (*r*: 0.53), IGFBP-2 (*r*: −0.49), intact IGFBP-4 (*r*: −0.40), total IGFBP-4 (*r*: 0.37), prealbumin (*r*: 0.56)
Intact IGFBP-3	STC2 (*r*: 0.39), ALS (*r*: 0.63), total IGF-II (*r*: 0.34), prealbumin (*r*: 0.51), weight SD (*r*: 0.22)
ALS	Total IGF-II (*r*: 0.33), IGFBP-2 (*r*: −0.34)
IGFBP-2	Total IGF-II (*r*: −0.39), prealbumin (*r*: −0.40)
Intact IGFBP-4	IGFBP-2 (*r*: 0.34), prealbumin (*r*: −0.51), retinol binding protein (*r*: −0.47)
Total IGF-II	Total IGFBP-4 (*r*: 0.26), BMI SD (*r*: 0.22), weight SD (*r*: 0.25)

Significant correlations (*P* < .05) were found between the analyte in first column and those in the second column. Abbreviations: ALS, acid-labile subunit; IGF, insulin-like growth factor; IGFBP, insulin-like growth factor binding protein; PAPP-A, pregnancy-associated plasma protein A; PAPP-A2, pregnancy-associated plasma protein A2; STC, stanniocalcin.

## Discussion

To the best of our knowledge, this is the first study to report circulating concentrations of pappalysins and stanniocalcins in children with T1DM. As expected, upon exogenous insulinization there was significant metabolic (HbA1c) and nutritional improvement (transferrin) during the following year. Other parameters showed a tendency to improvement, such as growth velocity and BMI, although this was not statistically significant, probably because they were already within the normal range at presentation. The impact of height at T1DM diagnosis has been much debated, with studies reporting children with T1DM being taller than their healthy peers [1, 30], but this was not confirmed by other studies [31]. One suggested explanation for the increased height of children at the onset of T1DM, or “accelerator” hypothesis [1, 30], involves the insulinopenia in the prediabetic phase that results in increased IGFBP-3 proteolysis, with a subsequent rise in the availability of IGF-I [10]. Herein, we confirm this increase in IGFBP-3 proteolysis; however, no relevant changes in circulating free IGF-I were observed, likely due to the data dispersion. In contrast to a previous study, we found no association between growth velocity after T1DM onset and beta-cell reserve (evaluated by C-peptide concentrations after treatment) [10]. A possible explanation for this is that most of our patients already had a normal stature at presentation and their growth velocity did not statistically differ after insulinization. Total IGF-I concentrations were lowest at diagnosis, when the highest HbA1c levels were obtained. This is in accordance with other studies in pediatric populations [11, 32], as well as the increase in free IGF-I over time following metabolic improvement with intensive exogenous insulin treatment [33].

**Figure 3.**
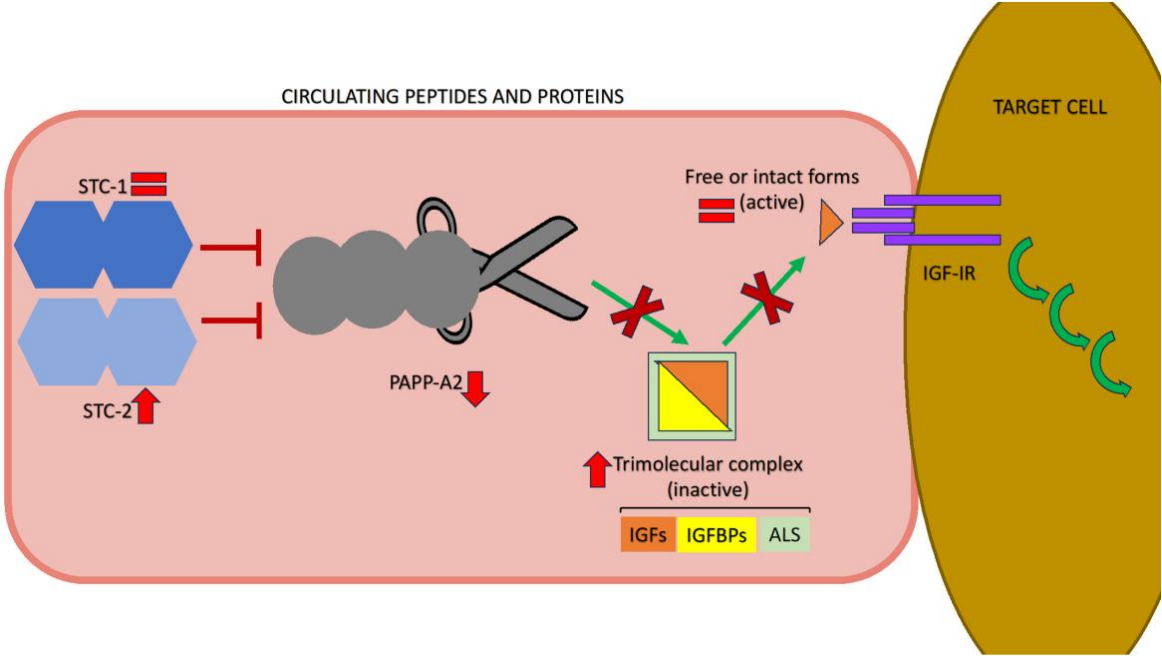
Schematic cartoon of the biochemical findings in the peripheral GH/IGF axis in children with T1DM. From T0 to T1 and T2, there is progressive increase in the concentrations of STC2, paralleled by decrease in PAPP-A2 and overall increase in most forms of total IGFs and IGFBPs. There is significant increase in intact IGFBP-3, but change is nonsignificant for other free forms. Arrows indicate the direction of change, if found; the equals symbols indicate the lack of relevant change in concentrations of analyte.

Significant modifications in STC2 levels were found during the study period, but not in STC1. We previously hypothesized that there is a more potent role of STC2 vs STC1 with regard to impact on linear bone growth [29], with STC2 also possessing other physiological roles in calcium-phosphate regulation, cell development, cytoprotection, and angiogenesis [25, 34], as well as affecting appetite and body weight regulation [34, 35]. Although a correlation was found between STC2 and body fat percentage in one cross-sectional human study [36], we observed no correlation with BMI, although there was a significant correlation with prealbumin, suggesting nutritional implication. The relationship between serum concentrations of STC2 and glucose metabolism remains uncertain. A study of 122 healthy adults failed to observe a relationship between these factors [36], which is similar to another study in adult T2DM patients [21]. However, another study in adults with T2DM found that those with the highest HbA1c exhibited the lowest expression of STC2 in platelets [22]. Moreover, in adults after Roux-en-Y, among those with a decrease in STC2, the levels of total IGFBP-4 correlated with an improvement in HbA1c, fasting glucose and insulin [25]. The decrease in PAPP-A2 over time coincides with the increase of most total forms of IGF, IGFBPs, and ALS, with no statistical change in the free forms (Fig. 3 schematically represents this study’s findings). In concordance with these findings, HbA1c negatively correlated with STC2 and positively with PAPP-A2. Metabolic improvement over time associates with an increase in the total forms of IGF-I and IGFBP-3, and it coincides with the described changes in STC2 and PAPP-A2. The underlying pathophysiological explanation of this remains unclear.

Several total forms of IGFBPs increased over time, including IGFBP-5, which is known to have a role in muscle growth and differentiation [37]. Unfortunately, discrepancies between IGFBP measurements across studies often occur, as many assays are not able to reliably differentiate between intact and degraded IGFBP. IGFBP-2 is reported to participate in growth regulation, body composition, and bone development [16] and to have antidiabetic effects [38]; however, we found no relevant modifications in its concentrations during the first year of insulin treatment or in comparison with the concentrations in healthy children; even though it has been described to be inhibited by insulin [39].

The association between PAPP-A and diabetes is inconsistent in clinical studies in adults, with statistical association between PAPP-A and prediabetes and T2DM reported in one study [23], but not in others [25-27]. During maintained hyperglycemia, TGF-β is reported to upregulate the expression of PAPP-A and IGF signaling [19]. However, here PAPP-A levels were not significantly increased and did not show relevant modifications over time. An explanation for this could be that PAPP-A has an active proteolytic action at the cellular level [15], in proximity to the IGF-I receptor (IGF-IR) in various target tissues, and therefore mainly influences IGF-I actions locally [15], with concentrations changing at the tissue level. Furthermore, PAPP-A concentrations may not represent modifications in its proteolytic activity, which has been previously reported to change under different physiological situations [40]. It is also possible that increased levels of STC2 inhibit the activity of PAPP-A [41]. Furthermore, our previous pediatric study showed that PAPP-A concentrations remain fairly constant during postnatal life in healthy children [29], as they appear to do during the first year after T1DM onset. Unlike in healthy individuals where no correlation was found between concentrations of STC1 and PAPP-A [29], correlation was identified in T1DM, of uncertain explanation given the small change overtime of the concentrations of both analytes.

IGFBP-4 is known to have roles in skeletal growth [42] and bone physiology [43], and in children with obesity, it positively correlates with fasting insulin concentrations [44]. Here, total IGFBP-4 significantly increased at 12 months, in unison with the rise in STC2, despite no significant modification in PAPP-A levels. PAPP-A was formerly considered the only protease to degrade IGFBP-4 [45], albeit metalloproteases other than the PAPP-As proteolyze IGFBPs [34]. This supports the hypothesis that at a cellular level STC2 blocks the degradation of IGFBP-4 through inhibition of PAPP-A activity [46].

Although PAPP-A, IGFBP-4, and STC2 are expressed in adipose tissue and are known to change with weight loss [25, 34], here no specific association with BMI was found. However, a negative correlation of IGFBP-4 with both prealbumin and retinol binding protein was found, while STC2 correlated with the nutritional marker prealbumin, suggesting a nutritional role of these factors.

Interestingly, no statistical differences were observed between sexes or when comparing prepubertal and pubertal concentrations of all the studied markers of the GH/IGF axis. The exception to the latter involves total IGF-II concentrations, which were significantly higher in prepubertal vs pubertal children, in contrast to a study where no changes were found during childhood [47]. This discrepancy is not yet fully understood.

Given that STC1 colocalizes with insulin in beta-cells [20], its suitability as a diabetes marker has been considered [21, 22]. However, we found minimal changes in its concentrations during follow-up, suggesting that it may not serve as a beta-cell reserve marker in children with T1DM. In adult T2DM patients, significant correlation was found between STC1, glycemia and HbA1c [22], but not here in our study of T1DM. This lack of correlation is logical, as in T1DM the insulin reserves are basically nonexistent in contrast to T2DM.

The main caveat of this study includes only measurement of circulating concentrations, as quantifying the activity of pappalysins and stanniocalcins in serum or at the cellular level [40] would provide further in-depth understanding of the physiology of the peripheral GH/IGF axis in these patients. However, we have recently published circulating levels of members of this axis in patients with anorexia nervosa and Prader-Willi syndrome [48, 49], with these studies indicating that indeed information on circulating levels can provide relevant information. Another limitation of this study is the lack of growth velocity prior to the diagnosis of T1DM and before starting insulin therapy (during a period of insulinopenia), to confirm the clinical implications of the findings in this study. Moreover, if 2 groups had been available (one poorly controlled and one with good glycemic control) to compare the circulating IGF system in both groups, further insight regarding the importance of glycemic control may have been concerned in the study; however, all patients had optimal metabolic control during the duration of the study. During the first year after T1DM diagnosis, most families become highly involved in the correct management of diabetes and, under these circumstances, growth should be minimally impacted. Thus, a more long-term study may be of interest, as often years after the onset of the disease, patients and/or relatives/caregivers become less strict with metabolic control, and this could impact growth. Additionally, the growth-promoting effect of insulin through the ubiquitously expressed IGF-IR [50], as well as IGF-IR signaling promoting insulin sensitivity [51], should be considered as possible growth enhancers.

## Conclusions

Our results indicate that implementation of insulin treatment after T1DM onset modifies various components of the circulating IGF system, including those of PAPP-A2 and STC2. The orchestrated interplay between the GH/IGF members, along with adequate metabolic control and nutrition optimization, due to correct insulinization, participates in promoting linear growth.

## Data Availability

Some or all datasets generated during and/or analyzed during the current study are not publicly available but are available from the corresponding author on reasonable request.

## Abbreviations

ALS: acid-labile subunit
BMI: body mass index
GH: growth hormone
HbA1c: glycated hemoglobin
IGF: insulin-like growth factor
IGFBP: insulin-like growth factor binding protein
IGF-IR: insulin-like growth factor I receptor
IQR: interquartile range
PAPP-A: pregnancy-associated plasma protein A (pappalysin)
STC: stanniocalcin
T1DM: type 1 diabetes mellitus
T2DM: type 2 diabetes mellitus
T3T: total triiodothyronine
